# Bone transport versus acute shortening for the management of infected tibial bone defects: a meta-analysis

**DOI:** 10.1186/s12891-020-3114-y

**Published:** 2020-02-06

**Authors:** Hongjie Wen, Shouyan Zhu, Canzhang Li, Yongqing Xu

**Affiliations:** 1 Department of Orthopaedic Surgery, The Fourth Affiliated Hospital of Kunming Medical University, Kunming, China; 2 Department of Radiology, The Fourth Affiliated Hospital of Kunming Medical University, Kunming, China; 3 Department of Orthopaedic Surgery, 920th Hospital of Joint Logistics Support Force, NO. 212 Daguan Road, Xi Shan District, Kunming City, 650021 Yunnan Province China

**Keywords:** Ilizarov technique, Tibial, Bone defects, Bone transport, Acute shortening

## Abstract

**Background:**

The treatment for infected tibial bone defects can be a great challenge for the orthopaedic surgeon. This meta-analysis was conducted to compare the safety and efficacy between bone transport (BT) and the acute shortening technique (AST) in the treatment of infected tibial bone defects.

**Methods:**

A literature survey was conducted by searching the PubMed, Web of Science, Cochrane Library, and Embase databases together with the China National Knowledge Infrastructure (CNKI) and the Wanfang database for articles published up to 9 August 2019. The modified Newcastle-Ottawa scale (NOS) was adapted to evaluate the bias and risks in each eligible study. The data of the external fixation index (EFI), bone grafting, bone and functional results, complications, bone union time and characteristics of participants were extracted. RevMan v.5.3 was used to perform relevant statistical analyses. Standard mean difference (SMD) was used for continuous variables and relative risk (RR) for the binary variables. All of the variables included its 95% confidence interval (CI).

**Results:**

Five studies, including a total of 199 patients, were included in the study. Statistical significance was observed in the EFI (SMD = 0.63, 95% CI: 0.25, 1.01, *P* = 0.001) and bone grafting (RR = 0.26, 95%CI: 0.15, 0.46, *P* < 0.00001); however, no significance was observed in bone union time (SMD = − 0.02, 95% CI: − 0.39, 0.35, *P* = 0.92), bone results (RR = 0.97, 95% CI: 0.91, 1.04, *P* = 0.41), functional results (RR = 0.96, 95% CI: 0.86, 1.08, *P* = 0.50) and complications (RR = 0.76, 95% CI: 0.41, 1.39, *P* = 0.37).

**Conclusions:**

AST is preferred from the aspect of minimising the treatment period, whereas BT is superior to AST for reducing bone grafting. Due to the limited number of trials, the meaning of this conclusion should be taken with caution for infected tibial bone defects.

## Introduction

The treatment for infected tibial bone defects can be a great challenge for the orthopaedic surgeon. The occurrence and progression of infectious bone defects of the tibia are often associated with severe wound infection, soft-tissue defects, vascular and nerve injuries, and joint dysfunction, rendering treatment difficult [1–6]. Most studies [1, 2, 7–9] recommend the Ilizarov technique to repair tibial bone defects because it has several advantages. First, infection can be strictly controlled. Second, this technique can tackle varying degrees of bone defects and restore a limb’s discrepancy to a satisfactory length. Third, bone defects and soft-tissue defects can be repaired at the same time. Fourth, it eliminates the necessity of bone grafts and donor site morbidity. The main treatment methods include BT and AST, and both methods have their advantages and disadvantages [6, 10–14]. It is still unclear which choice is better.

Currently, there are numerous comparative studies of these two techniques, but no meta-analysis on this topic has been published. The aim of the present meta-analysis was to compare BT and AST for the treatment of infected tibial bone defects and provide some useful suggestions for orthopaedic surgeons when facing such disease.

## Methods

### Meta-analysis principles

No ethical approval was required because we performed all the analyses based on previous studies. The present meta-analysis strictly followed the principles of the Preferred Reporting Items for Systematic Reviews and Meta-Analyses (PRISMA) statement [15]. It was prospectively registered in the PROSPERO registry (CRD42019133659).

### Search strategy

The following databases were searched by two individual investigators (WHJ and ZSY): the PubMed, EMBASE, Cochrane, Web of Science Library and Chinese databases, including the Wanfang database and the CNKI. Each database was searched up to 8 August 2019, with language restricted to English and Chinese. We performed the comprehensive literature search by applying the keywords of ‘bone transport’, ‘bone transportation’, ‘Distraction osteogenesis’, ‘ilizarov technique’, ‘acute compression and distraction’, ‘acute shortening’, ‘bone defects’, ‘non-unions’, ‘tibial’. Detailed search terms are provided in additional file 1.

### Study selection

The inclusion criteria were defined as follows:

(i) open tibial fractures with tibial bone and soft-tissue defects;
(ii) randomised controlled trial (RCT), retrospective or prospective trials;
(iii) age ≥ 16 years old;
(iv) managements were either bone transport or acute shortening/lengthening with Ilizarov circular external fixator;
(v) the data of eligible patient was complete.

Exclusion criteria were defined as follows:

(i) reviews, case reports, meta-analyses, editorial articles or letters;
(ii) duplicates of previously published papers;
(iii) studies that included children (< 16 years old).

### Data extraction

A standardised protocol based on comprehensive literature search was designed to extract eligible articles. The following outcome variables were extracted for pooled analysis: external fixation index, bone grafting, heal time, functional results, bone results and number of complications. In one study the external fixation index was reported in days/cm, which was converted to months/cm. Moreover, the relevant information of eligible articles was extracted: the first author, country, year of publication, total number, study design, bone defects, and journal reference.

### Quality assessment

Quality assessment of each enrolled study was evaluated by SYZ, CZL and HJW based on a modified version (nine-star scoring system) of the Newcastle-Ottawa Scale (NOS) for retrospective studies [16]. Studies with NOS scores above or equal to the median were considered high quality (low risk of bias).

### Statistical analysis

Two individual investigators applied Review Manager Software (version 5.3; Nordic Cochrane Centre, Copenhagen, Denmark) to conduct statistical analysis and produce relevant plots. Standard mean difference (SMD) was used for continuous variables and Relative risk (RR) for the binary variables. The 95% confidence interval (CI) of each variable was calculated and presented. Statistical heterogeneity among studies was analysed (I^2^ < 50%, *P* > 0.01 is the test standard of heterogeneity.). The random-effects model was applied when the heterogeneity between subgroups was high (I^2^ > 50%, *P* < 0.05); otherwise, the fixed-effects model was used. The *P* value was regarded as the standard to choose the processing model when the I^2^ value was inconsistent with the P value, When *P* < 0.05, difference was identified statistically significant.

## Results

### Included literature

**Fig. 1:**
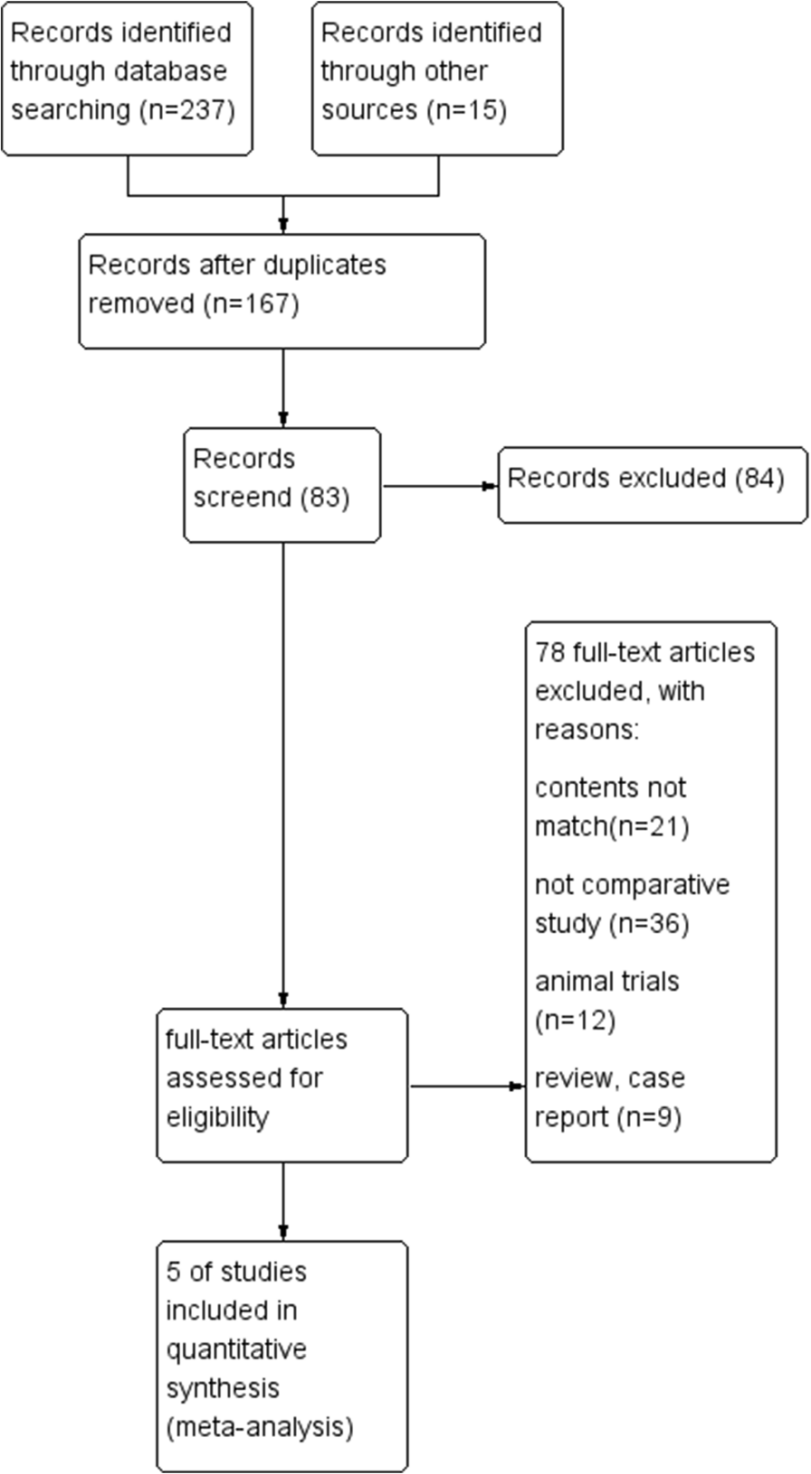
Flowchart diagram of the study selection

Concerning the study characteristics; a flow diagram showed the search procedure and the result was summarized in Fig. 1. A total of 252 related articles were searched, and 154 studies were excluded due to the title and abstract. Then, 78 articles were excluded from the 83 studies according to the inclusion criteria. Finally, five retrospective studies [6, 10, 12–14], including 199 patients, were included in the present study. In order to avoid heterogeneity, studies that only applied bone transport or acute shortening were excluded. In the meta-analysis, bone transport was set as the study group and AST as the control group. Tables 1 and 2 summarized the baseline characteristics of the eligible studies and patients.

**Table 1:** Characteristics of the eligible controlled trials

First author	Year	Country	Study design	Cases		NOS
				BT	AST	
Wu [14]	2017	China	retrospective	28	22	8
Kevin [13]	2017	Australia	retrospective	21	21	8
Levent [10]	2016	Turkey	retrospective	29	45	7
Yin [12]	2014	China	retrospective	18	13	6
Mahaluxmivala [6]	2005	UK	retrospective	6	6	6

*BT* Bone transport, *AST* Acute shortening technique, *NOS* Newcastle-Ottawa scale

**Table 2:** Baseline characteristics of the included patients

First author, year	Bone defect (cm) Mean value with ranges		Follow-up (months) Mean value with ranges		Gender (male/total)		Age (years Mean value with ranges	
	AST	BT	AST	BT	AST	BT	AST	BT
Wu, 2017 [14]	6.7(4.8–11.0)	6.4(4.3–10.0)	40.8(30–66)	40.8(30–66)	12/17	15/23	39.3(18–65)	38.8(16–67)
Kevin, 2017 [13]	5.8 (3–10)	7.0 (3–10)	20 (12–43)	31 (12–84)	18/21	18/21	39.2 (20–76)	38.2 (18–66)
Levent, 2016 [10]	5.9 (1–12)	5.3 (1–17)	55.6(12–66)	63(36–85)	38/45	20/29	34.8 (17–62)	37.6 (15–61)
Yin, 2014 [12]	6.3(4.5–9.0)	6.7(4–11)	28.8(16.8–54.5)	28.8(16.8–54.5)	11/18	8/13	39.3(18–65)	38.8(16–67)
Mahaluxmivala, 2005 [6]	4.6(3–6)	5.9(3–7.5)	>18^a^	>18^a^	6/6	5/6	36.5(26–54)	39.2(28–52)

*BT* Bone transport, *AST* Acute shortening technique; ^a^values are over certain age

Quality assessment of the included studies by using NOS for retrospective studies was presented in Table 3. The median score of NOS was seven. Therefore, among the five studies, three were considered of high methodological quality (low risk of bias); they scored ≥7 [10, 13, 14], whereas the other two studies [6, 12], which scored < 7, were therefore considered of low methodological quality (high risk of bias).

**Table 3:** Risk-of-bias assessment of the included studies, according to the modified Newcastle-Ottawa Scale (NOS)

NOS items / Study ID	Wu 2017	Kevin 2017	Levent 2016	Yin 2014	Mahaluxmivala 2005
Is the case definition adequate?	★	★	★	★	★
Representativeness of the cases	★	●	★	●	★
Selection of controls	●	★	●	●	●
Definition of controls	★	★	★	★	★
Study controls for the most important factor (i.e., age)	★	★	●	★	●
Study controls for the second important factor (i.e., sex)	★	★	★	★	●
Was the measurement method described?	★	★	★	★	★
Were the methods of measurements similar for cases and controls?	★	★	★	★	★
Non-response rate	★	★	★	●	★
Total Score	8	8	7	6	6

★was awarded when the respective information was available

● was awarded if the respective information was unavailable

## Results of the meta-analysis

### Bone union time

**Fig. 2:**
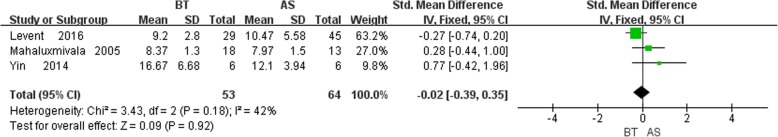
Comparison of bone union time between the BT and AST groups

In total, three studies [6, 10, 12] recorded bone union time, and no statistically significant heterogeneity was detected (*P* = 0.18, I^2^ = 42%), so the fixed-effects model was applied and the analysis results displayed that there is no statistically significant difference between control and study group (SMD = − 0.02, 95% CI: − 0.39, 0.35, *P* = 0.92). The results indicated that there is no difference in bone union time between two groups (Fig. 2).

### EFI

**Fig. 3:**
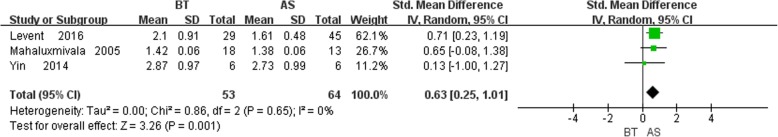
Comparison of external fixation index between the BT and AST groups

A total of three studies [6, 10, 12] reported an EFI, and no statistically significant heterogeneity was detected (*P* = 0.65, I^2^ = 0%). The fixed-effects model was applied to analysis the data and significant difference between the two groups was detected from the result (SMD = 0.63, 95% CI: 0.25, 1.01, *P* = 0.001). The results showed that the EFI of the AST group is lower than that of the BT group (Fig. 3).

### Bone grafting

**Fig. 4:**
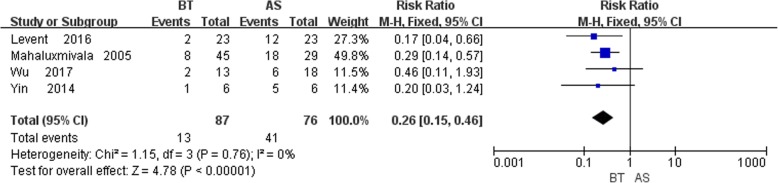
Comparison of bone grafting between the BT and AST groups

Overall, four studies [6, 10, 12, 14] recorded the bone grafting rate, and no statistically significant heterogeneity was detected (*P* = 0.76,I^2^ = 0%). The fixed-effects model was applied and significant difference between bone and AST group was detected from the result (RR = 0.26, 95% CI: 0.15, 0.46, *P* < 0.00001). The results displayed that the bone grafting rate of AST group is higher than that of the BT group (Fig. 4).

### Bone results

**Fig. 5:**
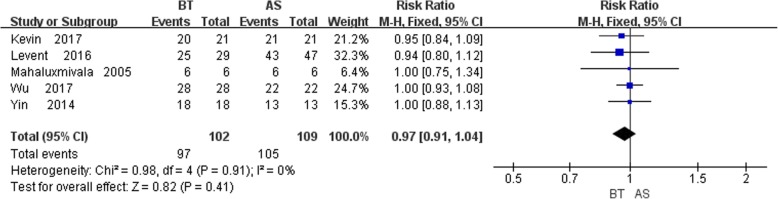
Comparison of bone results between the BT and AST groups

A total of five studies [6, 10, 12–14] recorded the bone results, and no statistically significant heterogeneity was detected (*P* = 0.91, I^2^ = 0%). The fixed-effects model was applied and no statistically significant difference between the two groups was found (RR = 0.97, 95% CI: 0.91, 1.04, *P* = 0.41). Therefore, statistical results displayed that there is no difference in bone union rate between control and study group (Fig. 5).

### Functional results

**Fig. 6:**
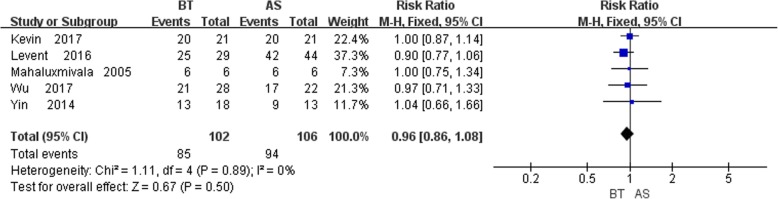
Comparison of functional results between the BT and AST groups

In total, five studies [6, 10, 12–14] described the functional results, and no statistically significant heterogeneity was detected (*P* = 0.89, I^2^ = 0%). The fixed-effects model was applied and the results displayed no significant difference between control and study groups (RR = 0.96, 95% CI: 0.86, 1.08, *P* = 0.50), indicating bone transport group was no better than AST group in functional results (Fig. 6).

### Complication

**Fig. 7:**
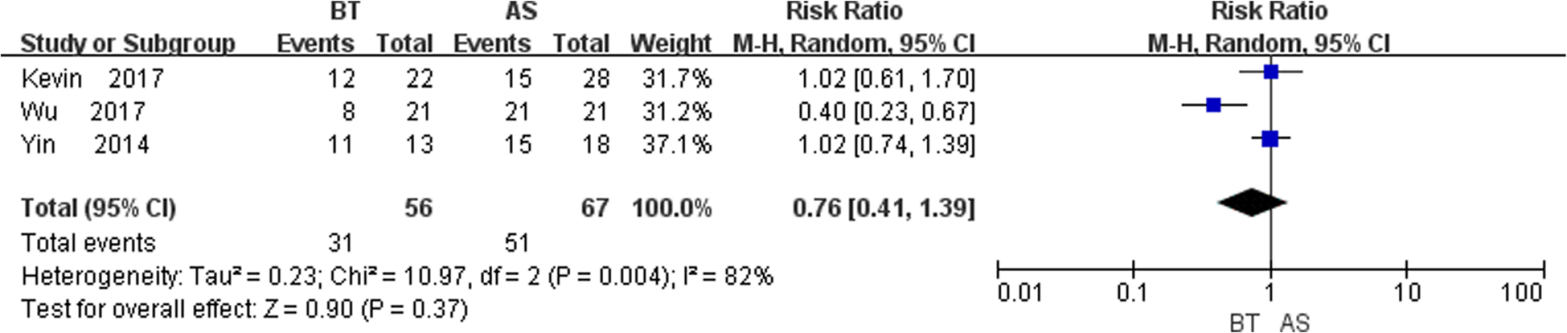
Comparison of complications between the BT and AST group

Complications were mentioned in three studies [12–14] and had statistically significant heterogeneity (*P* = 0.004,I^2^ = 82%). We performed pooled analysis by random-effects model and the results displayed that there was no statistically significant difference between the control and study group (RR = 0.76, 95% CI: 0.41, 1.39, *P* = 0.37), indicating bone transport group was no better than AST group in functional results (Fig. 7).

### Publication bias

**Fig. 8:**
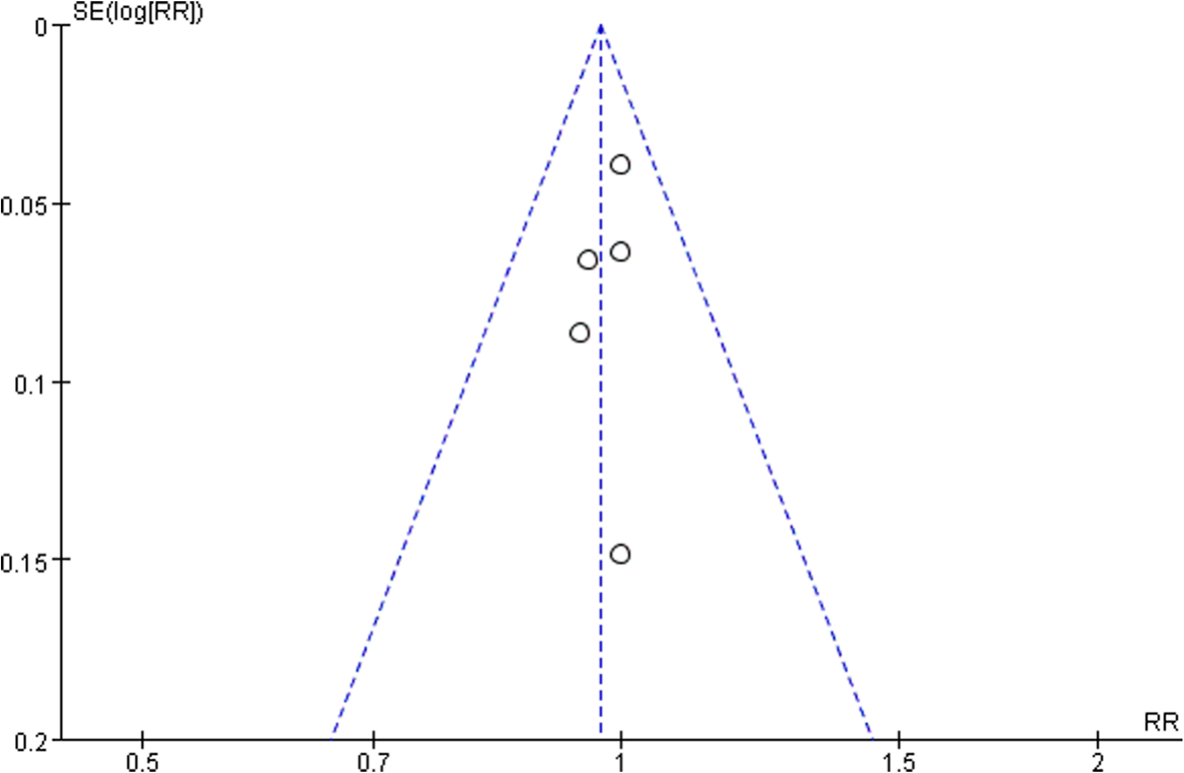
Funnel plot of the bone results of BT and AST groups. SE, standard error; RR, relative risk

A funnel plot according to bone results was produced to display whether there was publication bias. The result showed that the two sides of the funnel plot are roughly symmetrical, indicating low publication bias (Fig. 8). However, the number of included trials < 10, so the conclusion may not be completely accurate.

## Discussion

### Advantages and disadvantages of AST and BT from previous literature

Currently, Ilizarov reconstructions, the Masquelet technique, vascularised and non-vascularised bone grafts and bone substitutes are the main methods to treat tibia defects [2, 8, 9, 17–22]. However, bone transfer is the preferred technique for the treatment of infected tibial bone defects [1, 4, 23, 24]. Ilizarov reconstruction techniques include two main clinical treatment protocols: bone transport and acute shortening and gradual lengthening [11, 25]. Bone transport is a safe and reliable approach of tackling segmental tibia bone defect. It can simultaneously repair bone defect and soft-tissue defect. It has the advantages of quick wound healing, shortened treatment duration, less bone grafting and reliable treatment efficacy [26, 27]. However, postoperative complications are common, such as bone exposure and bone non-union usually along with axis deviation in long segmental bone transport, consolidation of newly formed bone is poor, delayed union or non-union can occur at the docking site, and pin track infection and screw loosening, stiffness of the knee and ankle joint foot drop can occur [5]. Many studies of the ASD technique have shown that it has obvious advantages and can significantly shorten the time of union [24, 28–30]. It reduces or closes the wound, effectively reduces the soft-tissue tension, and reduces the incidence of postoperative bone infection, bone exposure, osteonecrosis and soft-tissue necrosis; it is especially suitable for patients with large wounds [31–37]. However, according to two studies [38, 39], it may cause vascular and nerve injury and require more bone grafts and a limited shortening distance. At present, there are many comparative studies on bone transport and acute shortening technique in the treatment of infected tibial bone defects, but no conclusion has been reached. As far as the author knows, the present study is the first meta-analysis about the issue.

### Outcome analysis

#### EFI

The present meta-analysis showed that AST was superior to BT from the aspect of the EFI, which is an effective index to evaluate the treatment of bone defect and non-union with the Ilizarov technique, which is closely related to age, pathological characteristics, osteotomy position, elongation speed and bone defect length [40, 41]. Many studies reported that the EFI of the bone transport group ranged from 0.87 to 2.8 months/cm [4, 5, 23, 42] compared to 1.2–2.5 months/cm in the acute shortening group [24, 30, 32, 33, 35, 36, 39, 43]. In the present study, significant difference was detected in the two groups in terms of the EFI (*P* < 0.05) (Fig. 4), which means the EFI of the BT group was higher than that of the AST group. This result indicated the advantage of the AST group in treatment duration, which is consistent with the current mainstream literature.

#### Bone union time

A bone defect area is always filled with soft tissue just because bone ends cannot reach the docking site in time, which may consequently affect bone union time. The AS technique can bring forward and solve the problem of non-union because it shortens the duration of bone defect ends’ contact and performs bone grafting at an early stage [12]. Kemal et al. reported on a study of 24 patients with mean defects of 7.01 cm. They reported an average bone union time of 275.5 ± 70.6 days [5]. A study of 31 cases reported the mean time to union as 40.1 weeks (12.6–80.7 weeks) [32]. The mean healing index in another study was 30 days/cm [33]. MP Magadum et al. described the mean lengthening achieved as 10 cm, and mean union time was 6.3 months in a study of 27 patients with infected non-union and large bone defects in the tibia [30]. In the meta-analysis, no difference was detected in the two groups, according bone union time (*P* > 0.01). Some studies show that multiple-level bone transport can significantly decrease bone union time [44]. Results may also be affected by the different bone transport modalities used in the included studies. Some studies believe that docking site union is the key factor that affects the whole therapeutic time, and the AS technique is more advantageous in shortening the docking site union time [24, 28–30]. Therefore, the bone union time of the AS group may be shorter. However, bone union may be affected by many factors, such as the severity of the original injury and infection, the length of bone defect and other factors. In addition, the number of studies included was small, so the results should be critically considered.

#### Bone grafting

Four studies included in this study reported the bone grafting data, and the results showed that the difference between the two groups was statistically significant (*P* < 0.05), which means the AST group needed more bone grafting. At present, most of the research claims to perform bone grafting at the docking site to reduce the bone union time [25]. According to previous literature reports, the bone grafting rate of the BT group ranged from 14.3 to 40% [1, 23, 45] compared to 20–43% in the AST group, which is consistent with the present study [2, 11, 36].

#### Bone and functional results

All the eligible trials applied the Association for the Study and Application of the Method of Ilizarov (ASAMI) criteria to assess bone and functional results [30]. An excellent rate range from 64 to 83% in the BT group was documented, [4, 19] compared to 53–100% in the AST group [30, 34–36]. Kemal et al. reported bone union of 95.8% and 12 (50%) cases had excellent radiological results [5]. No difference was detected in the two groups, according to bone results (*P* > 0.01). This suggested that both groups were at the same risk for delayed union, malunion and non-union. Due to the limited number of references, there may be some bias in the results, so it is necessary to include more high-quality literature for further analysis and evaluation to draw a more accurate conclusion. All five eligible studies described the detail of functional results, and the result showed that a significant difference was found in the two groups (*P* > 0.01). Studies illustrated that excellent functional results ranged from 38 to 58% [4, 5, 19] in the BT group compared to 60–86% [30, 35, 36] in the AST group. The functional results mainly depended on professional guidance of functional exercise, prevention of needle penetration too close to the joint, adoption of methods to correct the existing ankle deformity and so on. Although AS has the advantage of earlier wound closure and avoiding a flap graft, the shortened tendon becomes relaxed and prone to foot drop [12].

#### Complications

Pin track infection and screw loosening are the most common complications in external fixation, and usually the final results will not be affected by these complications. In terms of reports, limb length discrepancy, permanent nerve and vascular damage, vascular crisis, re-fracture and newly formed bone infection are rare, and thorough debridement is the key step to controlling bone infection [4]. Studies reported that complications in the BT group ranged from 8.3 to 100% [1, 4, 23, 42] and 9 to 100% in the AST group [32, 33, 36, 37, 43]. Sarah et al. recommended the use of Doppler ultrasonography to assess distal pulses as necessary and to choose the appropriate shortening method according to the soft-tissue and wound condition [38]. Three studies published the data about complications, and one significant difference was found in the two groups according to the complications (*P* > 0.01). Because the included studies did not describe the types and detailed statistical data of the complications, this study could not carry out subgroup analysis, so there may be some bias in the results.

#### Strengths and limitations

To the best of our knowledge, this is the first meta-analysis to compare the safety and efficacy between bone transport and AST for the treatment of infected tibial bone defects. Moreover, low heterogeneity analysis and publication bias was detected in the meta-analysis. There were certain limitations in the present study. First, all of the eligible studies were retrospective studies, and the sample size was small; most studies were performed in a single centre, which may lead to a certain degree of bias. Therefore, part of the conclusions should be treated with caution. Second, the results may be affected due to the different inclusion and exclusion criteria and measurement indicators of each study. Third, the included literature lacked standardised and unified standards for the recording of variables, especially the external fixation index and bone union time, which resulted in many variables could not be combined for analysis. Fourth, in the five studies, further fixation after removal of external fixation was different, including nail, plate and plaster. The shortening methods were also disparate; immediate shortening or gradual shortening were applied in different studies, and the external fixation types included monolateral fixators and ring fixators. All of these selections may induce heterogeneity and impair the reliability of the conclusion. Therefore, further study based on large-size, multi-centre clinical RCTs, which apply unified and correct scoring system, evaluation indicators and random methods of blinding, is still necessary in the future for achieving higher-level evidence for clinical treatment.

## Conclusions

AST is preferred from the aspect of minimising the treatment period, but BT is superior to AST for reducing bone grafting. Due to the limited number of trials, the meaning of this conclusion should be taken with caution for infected tibial bone defects.

## Supplementary information

**Additional file 1.** The search strategies used in the platforms of PubMed and EMBASE.

## Data Availability

All data generated or analysed during this study are included in this published article.

## Abbreviations

95% CI: 95% confidence interval
AST: Acute shortening technique
BT: Bone transport
CNKI: China National Knowledge Infrastructure
EFI: External fixation index
NOS: Newcastle-Ottawa scale
RR: Relative risk
SMD: Standard mean difference
