# Supplementary material for: Bone transport versus acute shortening for the management of infected tibial bone defects: a meta-analysis
Source: BMC Musculoskelet Disord. 2020 Feb 6;21:80. doi: 10.1186/s12891-020-3114-y (PMC7006089; doi:10.1186/s12891-020-3114-y)
Supplement: Supplementary file 1 — Additional file 1. The search strategies used in the platforms of PubMed and EMBASE. [file 12891_2020_3114_MOESM1_ESM.pdf]

# Search term

**Search date limitation:through September 8, 2019**

**Pubmed**

(bone transport[TIAB] OR bone transportation[TIAB] OR distraction osteogenesis[TIAB] OR ilizarov technique[TIAB]) AND (shortening[TIAB] OR acute compression[TIAB]) AND tibia*[TIAB]

Records:115

**Embase**

(‘bone transport’:ti,ab OR ‘bone transportation’:ti,ab OR ‘distraction osteogenesis’:ti,ab OR ‘ilizarov technique’:ti,ab) AND (shortening:ti,ab OR ‘acute compression’:ti,ab) AND tibia*:ti,ab

Records:122
